# Effect of L-Glutamine Supplementation on Electromyographic Activity of the Quadriceps Muscle Injured By Eccentric Exercise

**Published:** 2013-06

**Authors:** Farhad Rahmani Nia, Esmail Farzaneh, Arsalan Damirchi, Ali Shamsi Majlan

**Affiliations:** 1 Department of Exercise Physiology Faculty of Physical Education and Sport Science, University of Guilan, Rasht, Iran; 2 Departments of Physical Education and Sport Sciences, Kermanshah Branch, Islamic Azad University, Kermanshah, Iran

**Keywords:** Eccentric exercise, Electromyographic activity, L- glutamine, Muscle injured

## Abstract

***Objective(s):*** The purpose of the present study was to examine the effects of L-glutamine on electromyographic (EMG) activity of the quadriceps muscle injured by eccentric exercise (EE).

***Materials and Methods: ***Seventeen healthy men (age: 22.35±2.27 yr; body mass: 69.91±9.78 kg; height: 177.08±4.32 cm) were randomly and double-blind study with subjects assigned to either an L-glutamine supplementation (n=9) or placebo (n=8) group. The subjects in two groups were asked to take three times during a week for 4 weeks. Each subject was screened for dietary habits before inclusion into the study. Participants performed 6 set to exhaustion eccentric leg extensions at 75% of 1RM and rest intervals were 3 min among sets. Pain Assessment Scale (PAS), EMG activity and range of motion (ROM) measurements were taken before exercise protocol and 24 and 48 hr afterwards.

***Results:*** There was no statistically significant difference between groups in perceived muscle soreness (SOR), ROM and EMG activity (*P <* 0.05).

***Conclusion:*** The results indicate that L-glutamine supplementation has no significant effect on muscle injury markers in between groups, although glutamine supplementation attenuated delayed onset muscle soreness (DOMS) effects in sup group.

## Introduction

DOMS and impaired muscle function are the common consequences of excessive EE (1). Many athletes use a variety of dietary supplements to improve their performance and minimizing risk of injures to provide them with an advantage over their opponent (2). Multiple roles of dietary protein and key amino acids such as glutamine create a variety of potential applications for hard-training athletes (3). Glutamine is the most abundant amino acid within the human body (4). During exercise, increases and decreases in plasma glutamine levels have been demonstrated and these variations are reflected by the type, duration, and intensity of exercise (5). Exercise induced muscle damage to muscle fibers resulting in an inflammatory response (6) and myofibrillar damage along the Z-band (7). Increase intramuscular glutamine levels have been directly linked to influencing muscle cell volume (8), which enhances protein synthesis, and increases muscle size. By increasing muscle mass, the contractile force of a muscle can be increased (2).

Hence, studies have demonstrated that by administering an L-glutamine supplement to mice through their drinking water, their skeletal muscle contractile forces were increased. With a greater contractile force, they conclude that the mice receiving the L-glutamine experienced greater muscle growth. Large muscles produce greater forces of contraction due to the increased number of myofilaments available to create a muscle contraction (2). During stressful event states, the consumption of glutamine in immunologic tissues and cells increases. This increase in consumption, coupled with enhanced utilization by other tissues, results in a demand for glutamine that outstrips supply (6).

On the other hand skeletal muscle injury induced by EE has been associated with SOR, decrease ROM following inflammation along with myofibrils disruption after EE (9) and changes in the EMG signal (7). Surface EMG is a technique for evaluating and recording physiologic properties of muscles at rest and during exercise. Additionally, some authors suggest that surface EMG can indicate muscle damage by selective recruitment of difference motor units. One popular measure of EMG frequency content is median frequency (MDF), the point at which the spectral power is divided into equal low and high-frequency halves and the mean power frequency (MPF) reﬂects the characteristics of frequency of EMG. MDF and MPF is a recommended variable for the study of muscle fatigue and damage (10, 11).

In a recent study, for example, Cruzat VF *et al* (2010) concluded that supplementation with free L–glutamine and the dipeptide alanyl-glutamine represents an effective source of glutamine, which may attenuate inﬂammation biomarkers after periods of training and plasma levels of CK and the inﬂammatory response induced by prolonged exercise in rats (4).

It is possible that exogenous supplementation of glutamine may decrease the severity of the inflammatory response, resulting in less muscle damage and possibly enhancing muscle recovery and also previous research projects on rats and prolonged exercise protocol, while eccentric resistance protocol is rarely considered. Therefore, the present research tries to investigate the effect of L-glutamine on EMG activity of the quadriceps muscle injured by eccentric exercise in untrained men.

## Materials and Methods

***Subjects***

Seventeen healthy college-aged untrained men between 19–25 years old were recruited. They had not performed regular resistance training for at least six months. Prior to initial testing, each subject was informed as to the experimental procedures, completed a personal/medical history form and signed an informed consent form before participating in the study. 

***Diet assessment***

Each subject was screened for dietary habits before inclusion into the study. Subjects completed 7-d diet records 1-d randomized in each week and 3-d during the training period. After 4-d diet record, participants received dietary counseling by a registered dietitian to ensure that the participants maintained their habitual dietary habits and ingested the supplement three times in week and assure adherence to all supplement and food instructions. Food diaries were analyzed for energy and macro/micronutrient content with NUTRITIONIST IV (Version 3.5.2).

***Supplementation***

This is a randomized; double-blind study with subjects assigned to either an L-glutamine supplementation (sup) (n=9) or placebo (pla) (n=8) group. The subjects in the sup and pla groups were asked to take three times in a week for 4 weeks. Subjects received 0.1 g/kg of L-glutamine (Glutamine; Karen pharma & food supplement. co. Iran) mixed with sugar-free lemonade or a placebo (maltodextrin with sugar-free lemonade). The subjects of both groups were taking the sup and pla after lunch during the 4 weeks. The sup or pla was mixed in ~500 ml of water. The subjects were asked to avoid physical activities during the study.

***Experimental design and exercise protocol***

During the week before the commencement of the study, subjects were given an orientation to the testing and initial measurements. Testing was conducted at the faculty of physical education physiology laboratories at Guilan University. Subjects were tested for body mass, body composition, and determining each subject’s one repetition maximum (1RM) in the leg extension exercise. On the first visit, subjects reported to the laboratory after 4 weeks. Assessment of EMG activity, ROM and muscle soreness was evaluated using the PAS then subjects performed exercise protocol. The exercise protocol was performed according to a modiﬁcation of a protocol described by Stock *et al* (12). Brieﬂy, exercise protocol was performed in order to induce muscle damage including leg extension (6 set × 75%1RM, to exhaustion) done by leg extension machine with an emphasize on eccentric portion and rest intervals were 3 min among sets. The same experimental procedure (PAS, EMG activity and ROM measures) was conducted 24 and 48-hr post exercise protocol. 

***Measurements***

*Muscle soreness*

Each subject was asked to evaluate his level of perceived muscle soreness (SOR) using a PAS. In this scale, 0 exhibits no pain and the score 6 indicates the maximal perceived pain, and should be choosen the number that corresponded to their perceived level of soreness.

*Electromyographic activity recordings*

The subject performed three maximal voluntary isometric contractions (MVIC) separated by 2-min rest. During each MVIC contraction verbal encouragement was provided. Surface EMG of the vastus lateralis (VL) and rectus femoures (RF) muscles was measured during MVIC; the participants were in a seated position with restraining, straps across the chest and hip with the right knee 90º ﬂexed. The Surface EMG was recorded bipolary by surface electrodes with an inter electrode distance of 20 mm from gastrocnemius at four sites on the right legs. Before electrode placement, the skin was lightly abraded. The electrodes were placed longitudinally on the muscle belly on each marked sites. EMG activity was assessment by MegaWin ME3000P8 device (MEGA Electronics, Ltd, Finland) and analyzed by MegaWin 2.01. EMG parameters have been used to evaluate muscle damage and recovery after EE. Amongst them, MDF, MPF have been used frequently in previous studies (10, 11, 13).

**Figure 1:**
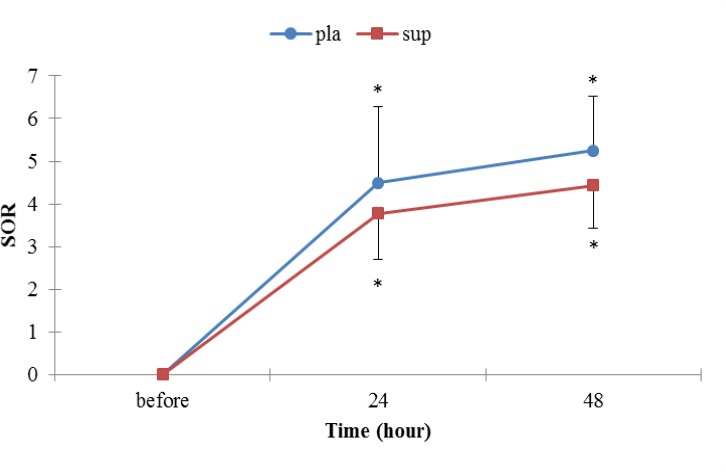
Muscle soreness for the placebo and supplementation groups after the EE.

* Denotes signiﬁcantly different from before EE values *P*<0.05. Data are mean ± SE

*Range of motion*

For the measurement of knee ROM, subjects laid prone situation with both knees fully extended. From this position subjects were asked to fully flex their right knee. The knee joint angle was determined by using a goniometer (jamar E2-Read, Clifton, New Jersey) and universal landmarks (lateral epicondyle of the femur, lateral malleolus and greater trochanter) to ensure alignment. 2 measurements were performed and the average was reported.

***Statistical analysis***

The statistical analysis was initially done using the changes in the One-Sample Kolmogorov-Smirnov test. All variables presented normal distribution. Two-way analysis of variance with repeated measurement was used to compare differences in MPF, MDF, SOR and ROM between sup and pla group at difference time points. Significant main effects were followed by bonferroni. Independent t-test was used to compare time points (before, 24 hr, 48 hr) in sup and pla group. Signiﬁcance was accepted when *P <* 0.05. SPSS 16.0 Statistical package was used for analyzing data.

## Results

Descriptive characteristic are listed in Table 1. There were no differences among groups for age, bodyweight, height, percent body fat and BMI. 

***Muscle soreness***

There was no statistically significant difference between groups in muscle soreness before, 24 and 48 hr after the EE (*P>*0.05), but the perceived pain was significantly different in each groups from before EE values. Perceived pain peaked 48 hr post-exercise in groups (Figure 1).

**Figure 2:**
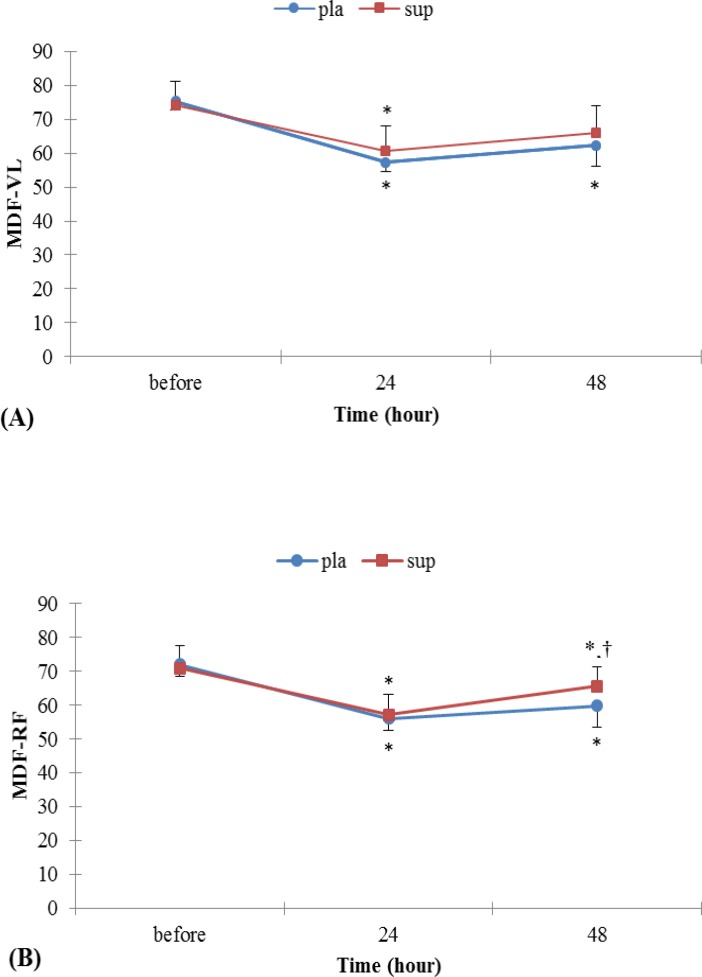
(A) Surface EMG index, MDF for vastus lateralis in leg extension before and after EE. (B) Surface EMG index, MDF for rectus femoures in leg extension before and after EE. * Denotes signiﬁcantly different from before EE values, † Denotes signiﬁcantly different from 24 hr after EE values *p<*0.05. Data are mean ± SE

***Electromyographic activity***

MDF and MPF for VL and RF were not different between groups.

*MDF*

As shown in Figure 2 A and B, there were significant time-by-pla group interaction effects for MDF for VL and RF muscles, but there was no significant difference in 24hr with 48 hr. Figure 2 A and B showed a significant decrease in MDF during MVIC after EE, which recovered afterwards. Whereas in sup group were significant time interaction effect for MDF for RF muscle but in VL muscle there was a significant difference only in 24 hr with before (*P<* 0.05).

*MPF*

**Figure 3:**
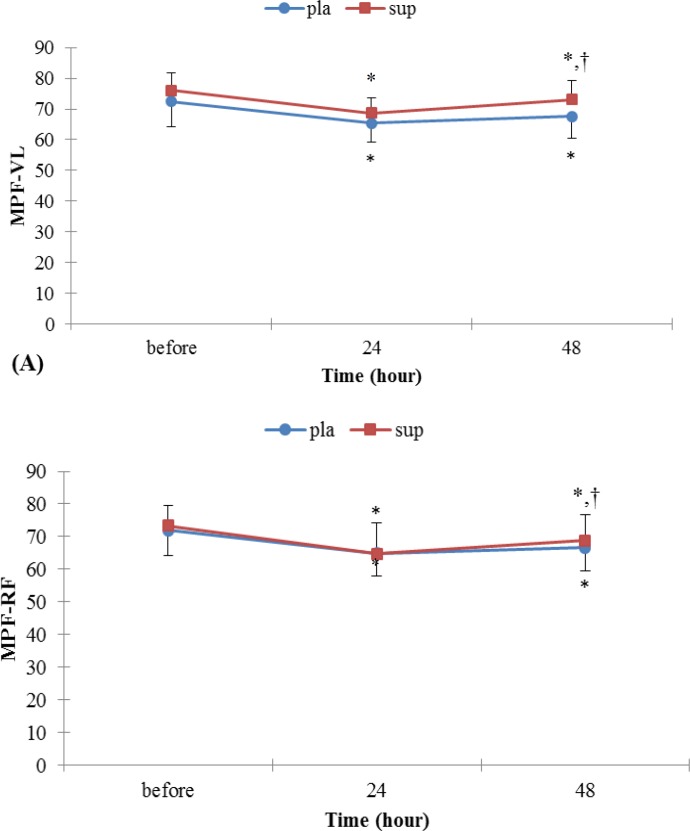
(A) Surface EMG index, MPF for vastus lateralis in leg extension before and after EE. (B) Surface EMG index, MPF for rectus femoures in leg extension before and after EE. *Denotes signiﬁcantly different from before EE values, † Denotes signiﬁcantly different from 24 hr after EE values *P<*0.05. Data are mean ± SE

As shown in Figure 3 A and B, the MPF in VL and RF muscle decreased during MVIC in each groups, but gradually returned towards the initial values in 48 hr after EE.MPF for VL and RF muscle in pla group with MDF for VL and RF muscle in pla group showed similar patterns. Nonetheless, there were significant time-by-sup group interaction effects for MPF for VL and RF muscles (*P<* 0.05).

**Table 1:** Individual Characteristics

Group	Age	Height	Weight	% Body fat	BMI
PLA	22.37±2.26	176.5±5.65	72.22±11.04	14.47±3.54	22.81±2.55
SUP	22.33±2.29	177.66±3	67.61±8.52	12.74±2.87	21.4±2.44

*P<*0.05. Data are mean ± SE

**Figure 4:**
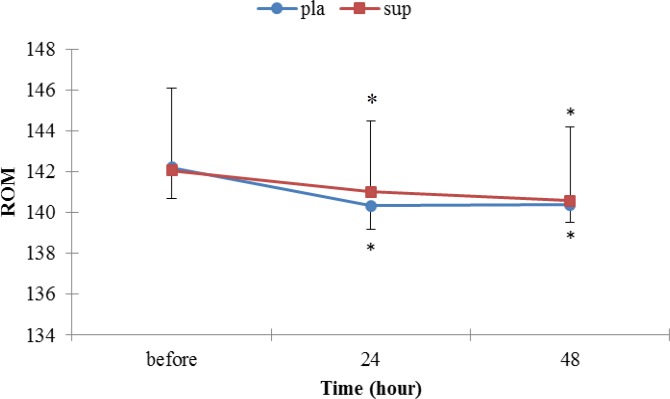
change in the knee ROM in pla and sup groups

***Range of motion***

There was no statistically significant difference between groups in ROM after the EE (*P>*0.05), but the ROM was significantly different in each groups from before EE values (Figure 4).

## Discussion

The purpose of this study was to determine Effect of 4 weeks of L-glutamine supplementation on EMG activity of the quadriceps muscle injured by eccentric exercise. 

It is generally agreed that there are two prominent signs of damage in a muscle immediately after it has been subjected to a series of eccentric contractions. There are the presence of disrupted sarcomeres in myofibrils and damage to the excitation–contraction (E–C) coupling system (14). In our study, the EE was successful in inducing muscle damage, which was evident from the significant change of dependent variables (ROM& perceive pain) of particular note is muscle sorenesess peaking at 48 hr post-exercise and also EE induced loss significantly of ROM 24 & 48 hr after EE as an indicator of passive muscle soreness (15), and there was no significant difference between groups, however sup group than pla group attenuated the exercise-induced decline in ROM. It is thought that this difference is due to effect glutamine supplementation in increased immunological cells.

Also, our data shows that MDF and MPF was decreased in the each groups for VL and RF muscle after 24 hr, but sup group greatly increase after 24 hr than pla group. This difference may be attributed to the effects of glutamine supplementation in increasing the amount of protein synthesis than by reducing degradation that can increase size fast twitch fiber (16). However, results of the present study is consistent with Cruzat VF *et al* (2010) (4) in within group, but this finding contradics the results attained between groups. This difference may have many reasons. One of them is related to methodology. Cruzat VF *et al* investigated the effect of the supplementation in rats to prolonged exercise, which makes differences between the present study and Cruzat. 

Nevertheless, reduce in MPF and MDF after the EE is consistent with Trevor C. Chen (17) and Linnamo V *et al* (13). This decrease could be explained by several mechanisms. For example, a possible reason is that some muscle ﬁbers were inhibited by ultrastructure damage after eccentric contraction, and this impaired the neuromuscular function (11). Also, elevated blood lactate concentration and increased proton (H^+^) accumulation may be partly responsible for the decrease in MDF. In the absence of blood lactate an impairment of the excitation-contraction coupling has been suggested to be responsible for the changes in MDF (13) and the selective injury of the fast twitch fibers after EE (11).

## Conclusion

The decrease of MPF and MDF, identified in the quadriceps muscle immediately after the EE, indicates a failure in the mechanism of muscle fiber contraction, probably associated with muscle fiber damage and from these results, the present study indicates that L-glutamine supplementation has no significant effect on muscle injury markers in between groups, although glutamine supplementation attenuated DOMS effects in sup group. 
